# Critical clinical situations in adult patients with Mucopolysaccharidoses (MPS)

**DOI:** 10.1186/s13023-020-01382-z

**Published:** 2020-05-14

**Authors:** Karolina M. Stepien, Anait K. Gevorkyan, Christian J. Hendriksz, Tinatin V. Lobzhanidze, Jordi Pérez-López, Govind Tol, Mireia del Toro Riera, Nato D. Vashakmadze, Christina Lampe

**Affiliations:** 1 The Mark Holland Metabolic Unit, Adult Inherited Metabolic Diseases, Salford Royal NHS Foundation Trust, Salford, M6 8HD UK; 2 Research Center for Children’s Health, Moscow, Russian Federation; 3 University of Pretoria, Pretoria, South Africa; 4 Vall d’Hebron University Hospital, Barcelona, Spain; 5 Anaesthetics Department, Salford Royal NHS Foundation Trust, Stott Lane, Salford, M6 8HD UK; 6 HELIOS Dr. Horst Schmidt Kliniken Wiesbaden, Wiesbaden, Germany

**Keywords:** Mucopolysaccharidosis, MPS, Adults, Anaesthesia, Multidisciplinary team, Surgical procedures

## Abstract

**Background:**

Mucopolysaccharidoses (MPS) are rare, inherited disorders associated with enzyme deficiencies that result in glycosaminoglycan (GAG) accumulation in multiple organ systems. Management of MPS is evolving as patients increasingly survive to adulthood and undergo multiple surgeries throughout their lives. As surgeries in these patients are considered to be high risk, this can result in a range of critical clinical situations in adult patients.

**Results:**

We discuss strategies to prepare for and manage critical clinical situations in adult patients with MPS, including supporting the multidisciplinary team, preoperative and airway assessments, surgical preparations, and postoperative care. We also present eight critical clinical cases (age range: 21–38 years) from four leading inherited metabolic disease centres in Europe to highlight challenges and practical solutions to optimise the care of adult patients with MPS. Critical clinical situations included surgical procedures, pregnancy and a thrombus in a port-a-cath.

**Conclusions:**

Individualised strategies to manage critical clinical situations need to be developed for each patient to compensate for the heterogeneous symptoms that may be present and the potential complications that may occur. These strategies should include input from the wider MDT, and be coordinated by metabolic specialists with expertise in the management of MPS disorders and surgery in adult patients with MPS.

## Background

The mucopolysaccharidoses (MPS) are a group of rare metabolic disorders caused by deficiencies of lysosomal enzymes that degrade glycosaminoglycans (GAGs) [1]. Across MPS disorders and severities the majority of organ systems may be affected, resulting in a broad range of acute clinical situations requiring input from a range of specialities [2–4]. Musculoskeletal, respiratory, cardiac, visual and auditory symptoms are common across all subtypes, while spinal cord compression and instability occur commonly in MPS I, II, IV and VI, and neurological problems occur in MPS I and severe MPS II, III and VII phenotypes [2–9]. New diagnostic technologies and therapeutic developments have meant that patients increasingly survive into adulthood [10–13]. The symptoms of MPS are progressive and are highly likely to require continued treatment throughout the patient’s life. Although enzyme replacement therapies (ERTs) are available for the management of some symptoms in some MPS subtypes, and haematopoietic stem cell transplantation (HSCT) provides a further treatment option [10, 14–18], patients with MPS often require multiple surgeries throughout their lives to manage symptoms and support optimal quality of life [4, 19]. Across the MPS disorders, surgeries include, but are not limited to, cardiac valve replacement, spinal decompression, tracheostomy, hernia repair, corneal transplant tonsillectomy, adenoidectomy, and insertion of ventilation tubes [2–5, 19, 20]. The combination of cardiac and respiratory dysfunction, spinal cord compression and instability, and anatomic abnormalities increases the risk of acute decompensation, requiring close surveillance pre-, peri- and post-surgery [4, 21, 22].

As patients with MPS reach adulthood, the responsibility for patient care is often transferred from paediatric to adult care teams, with the aim of allowing patients to become more independent and be more involved in treatment decisions if appropriate [23]. Although the transition process is a vital stage in supporting patients with MPS as they move towards increased independence, it is key to remember that patients have a progressive disease and the establishment of appropriate, individualised care plans in the adult setting is of great importance. Furthermore, an increasing number of patients with MPS are having children [24, 25]. This may impose additional life-threatening situations during pregnancy and caesarean sections, which are required to compensate for short stature and skeletal abnormalities [25].

As MPS disorders have historically been considered paediatric diseases, the patient’s adult care multidisciplinary team (MDT) may have less experience in managing the broad spectrum of potential critical clinical situations compared with paediatric colleagues. The continued involvement of paediatric specialists in the care of adults with MPS allows not only the provision of guidance and practical experience of the challenges of MPS, but also access to paediatric surgical equipment, which may be more suitable for adult MPS patients of small stature [4, 21, 23]. Even within an adult metabolic setting, the rarity of MPS disorders and the high risk and low frequency of the surgical procedures in patients with MPS may limit opportunities for MDTs to gain practical experience of treating adults with MPS. Indeed, a survey of 1900 anaesthetists found that only 34 of them had experience of adults with MPS [4].

The process of managing a critical clinical situation in MPS requires coordinated, expert input. This should be available from the point of determining if a particular procedure should go ahead, to carrying out a thorough preoperative assessment of the patient, to having a well-trained and prepared team available during the procedure, to understanding the potential complications and likely requirements of the patient during recovery. In this publication, we examine key factors that should be considered at each stage of managing a critical clinical situation in an adult patient with MPS, and examples of clinical strategies that could be used. We also present a series of cases involving critical clinical situations in adult patients with MPS, highlighting specific challenges that an adult care MDT may encounter and how these can be managed.

## Results

### Strategies to manage critical clinical situations in adults with MPS

Alongside collection of cases, healthcare professionals (HCPs) also provided key strategies that support the management of patients with MPS through critical clinical situations. These are summarised in Table 1.

**Table 1:** Strategies for management of patients with MPS through critical clinical situations

Strategy	Details
Involve all relevant specialities in surgical preparations and incorporate into the MDT (Fig. 1)	Coordinate through a metabolic specialist Incorporate paediatric HCPs if additional expertise is required Ensure the MDT is provided with expert information on patient needs and possible complications
Gain advice when developing standard operating procedures	Collate input across the MDT Incorporate information from guidelines, recommendations, publications and congresses
Monitor patient for progression of symptoms and development of adult-specific conditions (Multidisciplinary review and MPS Passport in Supplementary information)	Monitor complex symptoms throughout the patient’s life and new symptoms as they emerge Screen for common adult diseases, such as diabetes, cancer and hypertension
Discuss surgical procedures with patient and family well in advance	Allow patient and family to ask questions about surgery and choose where surgery is carried out Explain risks and benefits of proceeding with or not proceeding with surgery Present other therapeutic options and likely outcomes
Assess surgical risk and always carry out preoperative assessments (The preoperative assessment in Supplementary information)	Collate information from the MDT Balance risk of poor surgical outcomes with risk of no surgery Include input of patient and family preferences, and likely impact on quality of life Carry out all assessments required for general anaesthesia, even if a local anaesthetic is planned
Hold MDT meetings prior to surgery (The preoperative assessment in Supplementary information)	Ensure appropriate surgical expertise is available • Confirm surgical plans, and review current assessment results
Stabilise symptoms prior to surgery	Manage cardiac and respiratory dysfunction that may increase surgical risk
Individualise procedures and equipment for each patient (Surgical preparations in Supplementary information)	Prepare paediatric equipment and replacement devices (e.g. cardiac valves) for patients of small stature Adapt post-surgical management (e.g. post-surgical fluid volumes) for patients of small stature
Allow time for inclusion of additional procedures during surgery	Make surgeons aware of the potential need to manage complications associated with scarring from previous surgeries and/or MPS pathology Be prepared for: • Unsuccessful surgery • Problems with intubation • Haemorrhage • Post-surgical tracheostomy • Pain and/or discomfort • Infection
Anticipate need for emergency expert assistance during surgery	Ensure that paediatric anaesthetists, interventional radiologists and ENT specialists are aware of the procedure
Prepare a post-surgical care plan (Post-surgical care in Supplementary information)	Ensure that different options are available based on surgical outcomes and emergency procedures
Support patient and family after the critical clinical situation	Follow up patients with specialist members of the MDT Provide guidance on recovery and follow-up Ensure families support patients in following medical recommendations Provide prophylactic management and therapies to reduce negative impacts of ERT cessation
Keep up to date with expert opinions	Attend regional and national meetings Contact other experts for advice

*ENT* Ear, Nose and Throat, *ERT* Enzyme replacement therapy, *HCP* Healthcare professional, *MDT* multidisciplinary team, *MPS* mucopolysaccharidosis

#### The preoperative assessment

**Fig. 1:**
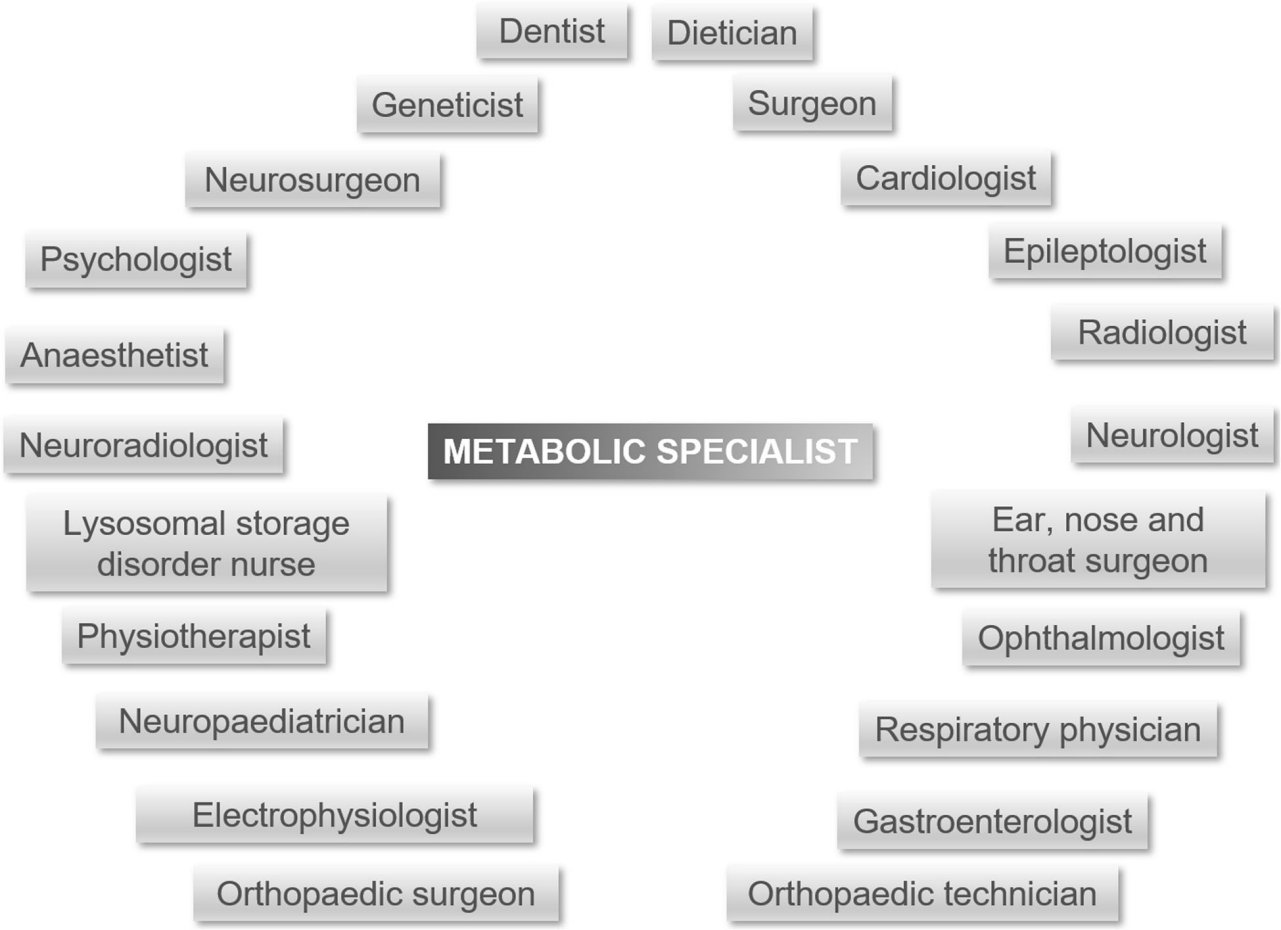
Key members of the MDT for patients with MPS. The MDT for patients with MPS consists of several HCPs. The main role of a tertiary adult metabolic centre is to coordinate the referral pathway to other specialists, arrange MDT meetings and follow up patients with MPS after procedures. In the UK model of care, a metabolic specialist coordinates referrals to other specialists and/or arranges preoperative assessments. Patients with MPS remain primarily under the care of a metabolic team, which works closely with the other specialists to provide the best possible care. The central coordinating position may be filled by any specialty with the skills required to manage the patient’s care plan among the other specialties involve

The importance of a thorough preoperative assessment was highlighted as being key in bringing together the members of the MDT who will be involved in managing the critical clinical situation, ensuring that all necessary information is obtained and an expert opinion is provided (Supplementary information: Multidisciplinary review and MPS Passport). The multidisciplinary review allows input from multiple disciplines, including metabolic specialists, cardiologists, anaesthetists, ear, nose and throat (ENT) surgeons, respiratory specialists, radiologists, neurologists and physiotherapists (Fig. 1). The outputs from this review are compiled into an MPS Passport, consisting of a clinical letter, images and videos, allowing any clinicians involved in the future management of a critical clinical situation, or clinicians located at different sites, to gain a thorough understanding of individualised recommendations and any associated challenges. This MPS Passport was developed by the Department of Anaesthetics at the Salford Royal NHS Foundation Trust, Salford, UK, and provides a reproducible, reliable approach for each patient. The multidisciplinary review is held as a single appointment, which is convenient for the patient and their carers, and also allows viewpoints from different specialities to be considered together. The images and videos can also be used to help patients and carers to understand the risks associated with any abnormalities of their airways or other investigation results.

As the standard tools and assessments used by anaesthetists may not be adequate for the assessment of patients with complex airways, such as those with MPS, a more thorough assessment involving an ENT consultant should also be carried out preoperatively (Supplementary information: Assessing the airways and preparing for emergency tracheostomy). Assessing cardiac risk may be challenging because of reduced mobility and chest deformities, which mean that tests that are used routinely in the general population, such as echocardiograms (ECHO) under stress, are not always suitable for patients with MPSs. Other suitable testing strategies may include transthoracic or transoesophageal ECHO, electrocardiogram (ECG), carotid intimal media thickness measurement and computed tomography (CT) coronary angiogram, although a short neck or inability to lie in the supine position due to bone deformities and lack of cooperation may make these studies less feasible for some MPS patients. In high-risk patients, and those expected to undergo long surgeries, intra-arterial blood pressure monitoring may be required.

##### Neurological considerations

Neurological symptoms in MPS disorders, such as epilepsy, impaired cognition and behavioural symptoms, are associated with accumulation of the GAG heparan sulphate, and these symptoms may become apparent or worsen as the MPS disorders progress and patients reach adulthood [9, 26–28]. The seizure threshold may be affected by anaesthetic agents and is managed by continuous intravenous AEDs administration during surgical procedures [29]. Aside from being used to treat epilepsy, antiepileptic drugs (AEDs) may also be used to manage behavioural symptoms, such as aggression and low mood [30], and plans will need to be made to ensure appropriate medication can continue during recovery. Electrolyte imbalances should be corrected in patients’ blood prior to and monitored after the surgery, as electrolyte disturbances are common causes of confusion in the hospital setting [31].

##### Patients undergoing transition to adult care from a paediatric setting

The complex surgical procedures in MPS disorders require clinical expertise that can be limited in the adult care setting [23]. Therefore, if an adult patient has not yet completed transition, and is still under some level of paediatric care, the MDT can assess if it would be most appropriate for the patient to remain in the paediatric setting during the perioperative and recovery periods, and for the surgery to be conducted by a paediatric team. If a patient develops an acute illness during the transition period, the MDT often decides that it is in the best interest of the patient to stay under the paediatric service while the critical clinical situation is resolved.

##### Engagement with the patient and family

The MDT must also prepare the family for any potential complications and negative surgical outcomes, such as an unsuccessful procedure and the risk of death during or after the procedure (Supplementary information: Discussing procedures with the patient and family).

#### Supporting the multidisciplinary team

Key members of the MDT and considerations for constituting the MDT are shown in the Fig. 1 and Table 1 respectively. HCPs with specialist knowledge and experience in paediatric MPS can provide guidance on or even undertake specific tasks. Furthermore, expertise may also be sought from external centres, and indeed HCPs may travel to provide practical support during procedures, or virtual MDTs may be established to allow input from multiple external experts. Although virtual MDTs do provide opportunities for geographically distant experts to be involved, this must be balanced with these experts having a less in-depth historical knowledge of the patient and their pre-surgical assessment results. Patients may also be referred to an expert in a distant centre, but this may delay decision-making and can cause distress if patients need to travel long distances or be assessed by unfamiliar HCPs. However the MDT is structured and supported, it should be ensured that specialists with experience of MPS either perform or provide guidance on the procedure [32, 33].

#### Specialised surgical equipment and surgical preparations

A full review of equipment and assessment results by the surgical team and an MPS expert prior to surgery will ensure that all requirements are in place (Supplementary information: Surgical preparations). Access to intraoperative monitoring options should also be investigated before surgery is performed. To avoid spinal injury, sensory injury with dysesthetic pain, and/or loss of proprioception, it is critical to maintain a neutral neck position during all surgeries, including during intubation and extubation [33]. Motor evoked potential and somatosensory evoked potential measurements may both be used to assess the risk of ischaemia and paraplegia, and are key in managing surgical risk in MPS patients with complex spinal abnormalities [34], although these options may only be available in specialised spinal and neurosurgical units. The requirement to access this equipment, and the need to work alongside experts able to interpret and promptly respond to the results, reinforces the need to carry out surgery in MPS in expert centres with specialist teams either carrying out or providing close guidance on the procedure [32, 33].

##### Cochlear implants

Patients with cochlear implants should not undergo procedures involving diathermy in the head and neck regions, although surgeons may not always be aware of this [35], and it is key that all members of the surgical team should be informed of any factors that would require a divergence from standard surgical procedures.

##### Baclofen pumps

A very small number of patients may also have implanted intrathecal devices to deliver baclofen to treat spasticity and dystonia, although these have not been shown to be effective in all MPS. The clinical experience of baclofen use in MPS patients is limited to few cases and the benefits were variable [36–38]. Complications associated with baclofen pumps are a frequent occurrence, and repeated surgeries are often required to manage these complications [36], which typically involve infections or mechanical failures.

#### Post-surgical plans

The recovery of the patient is also a complex, multidisciplinary process, which needs to be responsive to the outcome of surgery and any emergency procedures that were needed. Due to intubation during surgery and the accompanying soft tissue manipulation, patients may also be at risk of oedema of the airways. As this is frequently coupled with other respiratory features of MPS, post-surgical observation in an intensive care unit is a key stage of the initial recovery period [32, 33], and a place in such a unit should be available for all MPS patients in the 24 h following surgery. Feeding plans need to adapt as the patient recovers, and individualised physiotherapy requirements should be in place to support the patient’s rehabilitation (Supplementary information: Post-surgical care).

##### Post-surgical ERT

For the first 4 weeks post-surgery, it is recommended that ERT infusions are not provided, which is due to several key factors. First, the risk of anaphylactic reactions to ERT during sepsis or fever is higher; recommendations for MPS IVA, and safety information for ERT for MPS I state that infusions should be avoided in this acute post-surgical setting as patients may experience sepsis or fever during recovery [32, 39]. Second, adverse events associated with ERT can include fever and chills [14, 32, 33], and if ERT were provided soon after surgery, determining the cause of such symptoms as ERT-related or surgery-related would be challenging, leading to difficulties in managing the symptom. Third, staff in the post-surgery intensive care or recovery setting may not be familiar with administering ERT infusions and the precautionary procedures that should be carried out.

Continuation of ERT during longer periods of hospitalisation is likely to be affected by centre-specific and national circumstances. For example, in the UK, ERT is subject to additional costs for inpatients. In Germany, patients can continue ERT as outpatients, but coverage for inpatient ERT costs need to be negotiated with insurance companies. In contrast, patients in Russia can continue their infusions as inpatients. In Spain, there are no cost implications for continuing ERT for inpatients, although, in some regions, decisions to maintain or stop ERT would be taken by a specialist lysosomal disorders treatment committee. Due to this variation, an understanding of any restrictions is vital, and plans for infusions during the recovery period need to form a key part of the post-surgery strategy.

##### Post-surgical cognitive function

Aside from the physical aspects of a patient’s health status, the MDT must also consider the mental health and cognitive abilities of the patient. Several MPS disorders are associated with cognitive deficits, behavioural issues and epilepsy that need to be considered when assessing the patient’s recovery or their ability to communicate that they are in pain [4, 40]. Postoperative delirium is associated with a poor prognosis, and has been associated with baseline cognitive function, and cardiac and emergency surgeries, so it should be kept in mind when monitoring adult MPS patients post-surgery [41]. Postoperative cognition may also be affected by the use of some sedatives [42], analgesia in some populations [43], dehydration [44], preoperative infection [45], and some AEDs [46]. In MPS additional care should be taken to ensure that the patient is appropriately assessed, and that post-operative cognitive changes do not impact further on any pre-existing neurocognitive deficits. Plans to continue AEDs need to be in place, potentially with input from a neurologist, if the patient’s usual medication would interact with other perioperative drugs or a different method of AED administration is needed [29].

### Cases

Critical clinical cases and their specific challenges are summarised in Table 2. These cases illustrate examples of the types of challenges that might be encountered during critical clinical situations in patients with MPS but should not be considered exhaustive. Challenges associated with these surgical cases and critical clinical situations, and resolutions to these challenges, are shown in Tables 3 and 4, respectively.

**Table 2:** Critical clinical case details

Case	Patient characteristics	Critical clinical situation	Key team members	Hospital setting, preparations and management	Recovery and outcome
1 – cardiac valve replacement	Female patient with MPS VI, aged 34 years	Aortic valve replacement	Adult metabolic consultant, lysosomal storage disorder nurse, cardiologist, congenital heart disease specialist, cardiothoracic surgeon, paediatric and adult anaesthetist, ENT specialist, and respiratory consultant	• Specialist cardiothoracic theatre. Clinicians had expertise in congenital heart disease and previous experience in patients with MPS • The team was prepared for risk of unsuccessful surgery, problems with intubation, bleeding, cardiac arrhythmias and post-surgical tracheostomy	• After surgery, the patient was treated with warfarin and spontaneous ventilation. A nasogastric feeding tube was used because of a tracheostomy • Considered to be ‘good’ in this patient, as her tracheostomy was removed after 8 days and she was able to walk with support 14 days post-surgical • Follow-up by metabolic and cardiac specialists every 6 months
2 – spinal stenosis	Female patient with MPS VI, aged 21 years	Surgery to correct spinal stenosis and occipital spondyloses, involving installation of a halo device, laminectomy of the C1 vertebra, and resection of the foramen magnum	Neurologist with experience of MPS, neurosurgeon, geneticist, cardiologist and anaesthetist	Hospital specialising in orthopaedics	• Without complications over a 2-week period in hospital with physiotherapy support • Follow-up by MDT
3 – spinal stenosis	Female patient with MPS IVA, aged 21 years	Surgery to correct spinal stenosis in the cervical region	Metabolic specialist, orthopaedic surgeons, radiologist, neurosurgeons, anaesthetists and intensive care doctors	Hospital specialising in orthopaedics	• Without complications but the patient required physical rehabilitation during recovery because of muscular atrophy • Follow-up by a neurosurgery unit, with rehabilitation arranged through general practice
4 – corneal transplant	Male patient with MPS VI, aged 22 years	Corneal transplant – deep anterior lamellar keratoplasty	Adult metabolic consultant and nurses, ophthalmologist, adult specialist in corneal transplant, paediatric anaesthetist with expertise in MPS	• The team was prepared for pain, discomfort, infection and post-surgical haemorrhage • Surgery was performed under local anaesthetic • Although a local anaesthetic was planned, a full cardiac and respiratory assessment was conducted in case general anaesthesia was required	• As expected, and the patient could see shortly after the procedure • Discharge within 24 h, and, along with his family, was advised on how to prevent infection and injury • Follow-up in ophthalmology and metabolic clinics every 6 months
5 - pregnancy	Female patient with MPS I, aged 24 years	Pregnancy, birth and infant care	Obstetrician with expertise in inherited metabolic disorders, metabolic consultant, lysosomal storage disorder nurse, gynaecologist, midwife, general practitioner, cardiologist, genetic counsellor, anaesthetist and ophthalmologist	• Caesarean section planned for 38 weeks • The team was prepared to support the patient in caring for the infant as skeletal deformities and respiratory problems may have a negative impact on carrying the child and breastfeeding	• Baby born by uneventful spontaneous labour with epidural anaesthesia at 29 + 5 weeks • The patient developed mitral valve disease and underwent valve replacement and was treated with warfarin after surgery • She became pregnant again at this stage but, because of the teratogenic effect of warfarin, had a miscarriage
6 – thrombus development in a venous access device	Male patient with MPS II (Hunter syndrome), aged 26 years	Thrombus in a port-a-cath and change of venous access device needed	Metabolic consultant, lysosomal storage disorder nurse, infusion nurse, intravenous team, interventional radiologist, neurosurgeon, ENT consultant and anaesthetist	• Thrombus resolved using warfarin • The team was prepared for infections, further thrombi, blocked lines, and supporting the patient and family to manage the inconvenience of flushing access devices	• A Hickman line was inserted as a permanent solution for venous access • He was followed up in the adult care ssetting every 6 months
7 – complex continuous symptom management	Male patient with MPS II (Hunter syndrome), aged 33 years	• Respiratory, cardiac, neurological, gastrointestinal, skeletal, optic and dental symptoms • Multiple surgical procedures including adenoidectomy, tonsillectomy, T-tube insertion, inguinal and umbilical hernia repair, mastoidectomy, wrist surgery, dental surgery, hip replacement, tracheostomy, appendectomy, carpal tunnel decompression, two port-a-cath insertions, and a cardiac valve replacement • Recurrent respiratory infections and otitis, hepatosplenomegaly, concentration difficulties, endocarditis, and craniocervical stenosis	See Fig. 1	Managed by a metabolic adult care physician with expertise in MPS, based in a paediatric unit	• Patient now requires a hip replacement, but because of previous complications with airway management during surgery, this particular issue is managed through pain relief and use of a wheelchair • The patient requires glasses and hearing aids and has been prescribed medications for cardiac dysfunction
8 – complex continuous symptom management	Female patient with MPS I, aged 38 years	• Motor delay, kyphosis, hip problems and pain, recurrent respiratory infections, otitis, diarrhoea, short stature, joint contractures, back pain, aortic valve insufficiency, craniocervical stenosis, severe visual loss, and loss of sensitivity in the first three fingers of both hands • Cardiac valve replacement and spinal cord decompression	See Fig. 1	• Managed by a metabolic adult care physician with expertise in MPS, based in a paediatric unit • Very narrow airways, so anaesthetic equipment included paediatric intubation tubes that would not have been available in an adult hospital	Symptoms managed through ERT administered in an adult dialysis ward

**Table 3:** Challenges and resolutions associated with surgery

Challenges	Resolutions
**Cardiac valve replacement**	
• Initial intubation resulted in high CO_2_ pressure • Nasal intubation via right nostril also resulted in high CO_2_ pressure	• Paediatric and adult anaesthetists with experience in MPS disorders present • Intubation via left nostril successful
• Patient’s short neck made central line insertion difficult	• Ultrasound-guided central line insertion by paediatric anaesthetist
• Pericardial adhesions from previous mitral valve replacement surgery at the age of 24 years • MPS-associated valvular pathology	• Adhesions removed • Fibrous tissue, calcification and GAGs removed from mitral valve
• Physically small patient	• Paediatric catheters used to remove excess blood from ventricles • Smallest adult replacement valve used (size 19 mm CarboMedics Top Hat® mechanical prosthesis)
• Tracheostomy required because of narrow trachea, but difficult for paediatric and adult anaesthetists to perform	• ENT surgeon assisted • Pre-surgery 3D CT of chest and trachea, and fluoroscopy results were used to identify optimal site
**Spinal decompression**	
• No cardiology expertise in hospital performing surgery^a^	• Medical files provided by the treating doctor • Surgeons discussed surgery with treating doctor to understand MPS-specific requirements
• Patient and family did not wish ERT to be interrupted by surgery^a^	• ERT infusions arranged to occur during recovery at hospital performing surgery
• Patient had a short stature and restricted respiratory function^b^	• Neurosurgeon had extensive experience in paediatric patients
**Corneal transplant**	
• High cardiovascular risk	• Pre-surgery cardiac and respiratory function tests
• Risk that patient may not tolerate procedure or epithelium may be pierced	• Make preparations in case general anaesthesia is required
• Risk of graft rejection	• Endothelium preserved, resulting in reduced risk

^a^Case 2. ^b^Case 3

**Table 4:** Challenges and resolutions associated with critical clinical situations

Challenges	Resolutions
**Pregnancy**	
• Breathlessness and oedema progressed as ERT stopped at 3.5 weeks of the pregnancy	• Regular monthly obstetrics appointments • Regular cardiology, ophthalmology and anaesthetic appointments • Monthly fetal ultrasound scans
• Spinal support required during pregnancy	• Body corset worn by patient
• Neonatal child had squints, jaundice and respiratory difficulties	• Neonatal intensive care for 8 weeks • Supportive ventilation
• Help required caring for the baby for the first year because of joint restrictions in the hands	• Baby fed with expressed milk and formula • Support provided by patient’s family • Increased frequency of health visitor appointments • Appointments with occupational therapist
• Chest infections more frequent and forced vital capacity reduced, as ERT cessation continued during breastfeeding	• Antibiotics prescribed once bacterial infection confirmed • Contraindications confirmed with pharmacist
**Maintaining ERT administration after thrombus development in a venous access device**	
• Worsening breathlessness due to obstructive sleep apnoea	• Continuous positive airway pressure at night to resolve obstructive sleep apnoea prior to surgery to remove port-a-cath • Assessed by neurosurgeon, ENT consultant and anaesthetist prior to surgery
• After port-a-cath removal, patient received ERT by peripheral access, leading to reduced quality of life • Port-a-caths are usually reserved for paediatric patients	• Hickman line inserted
• Hickman line insertion resulted in patient distress	• Consider general anaesthesia for this procedure in patients with MPS
• Risk of infection with Hickman line • An adult Hickman line was required for an appropriate diameter, but as the patient is short, the line is relatively long, increasing infection risk	• Sterile dressings were changed frequently, and the line flushed prior to ERT • Patient and family educated on managing Hickman line and infusions
• Patient travelling shortly after procedure	• Sutures left in until patient was able to return
**Complex continuous symptom management**	
• Wide range of symptoms experienced, and surgeries and treatments required	• Adult care specialist has extensive experience of MPS and makes personal contact with the MDT to explain the requirements for each surgical procedure • Continued monitoring of symptoms that are life-threatening or may affect quality of life
• Airway management during extubation^a^ • Caused by swelling • Progressive dyspnoea developed after tracheostomy tube removal • Tracheal stenosis developed	• Emergency tracheostomy • Oxygen support required on some occasions • Assess need for all future surgeries
• Surgical management^b^	• Procedures can be carried out in a paediatric hospital that has appropriately sized equipment available and expertise in MPS
• Organisation of ERT infusions^b^	• Carried out by adult care clinicians in a dialysis ward

^a^Case 7. ^b^Case 8

## Discussion

This publication presents a range of challenges that may occur while managing critical clinical situations in adults with MPS. These challenges can be prepared for and resolved through practical steps and strategies, as outlined in Table 1 and the Supplementary information. Practical experience of managing critical clinical situations is often restricted to a limited number of specialist metabolic centres. Although some situations can be planned for in advance and the necessary expertise sought prior to the situation, for emergencies and time-limited procedures an awareness of MPS-specific requirements is needed to support HCPs in their treatment decisions.

In centres with experience of surgery in adults with MPS, there are opportunities to share this experience through providing advice and guidance, and sharing templates for preoperative assessments. It may even be an option for patients to undergo surgical procedures in these experienced centres, although this may limit the spread of knowledge if only clinicians from experienced centres are involved. Indeed, even experienced centres should seek to broaden their knowledge, as limited patient numbers mean that even an experienced centre may be managing small numbers of patients.

In adult metabolic centres with limited experience of MPS, the key is to seek out guidance and gain a full understanding of the challenges that are likely to be encountered. For example, difficult intubations because of narrowed airways are major factors in deferring complex surgeries [47]. Intubation difficulties are estimated to occur in 25% of patients with MPS, so the MDT may wish to take additional steps prior to this procedure to be fully prepared if initial procedures do not go ahead as expected. It should be emphasised that recommendations state that surgical procedures should be carried out by, or under the guidance of, MPS experts in centres with intensive care units, and some clinicians will have to assess if they have the necessary expertise and facilities at their centre to manage the challenges of surgery in patients with MPS [32, 33].

Coordination of MPS care is particularly important as patients with MPS are at high risk of surgical complications and knowledge of these is the key to optimising each procedure [4, 5, 21, 22]. A metabolic specialist should also be involved in decision-making with regard to the potential benefits and risks of surgery. While some patients may accept the risk of an additional post-surgical procedure, such as a tracheostomy, and are willing to undergo the procedure, others seem to develop tolerance to their stable and not troublesome symptoms and learn how to live with them. Because of the multi-organ involvement in MPS disorders and the potential for adverse impacts on surgery, where possible, symptoms should be resolved or stabilised before surgery is carried out.

Guidance on surgical procedures in MPS is already available, highlighting the importance of experienced team members and thorough preoperative planning [2–5, 22, 47, 48], and should be incorporated into standard operating procedures when possible.

In terms of managing pregnant patients, many patients will undergo a planned Caesarean section. This may be because of either small maternal pelvis size or the position of the baby, but it is also important to consider the risk of a patient with MPS requiring an emergency general anaesthetic [25]. Conversely, some patients might have increased intracranial pressure or hip dysplasia meaning that an epidural would be inappropriate. As with other surgery types in patients with MPS, recovery may be prolonged and a full preoperative assessment by an experienced MDT is needed. Plans should be in place for babies of mothers with MPS to be delivered in high-risk maternity units. Throughout pregnancy, some patients with MPS have been reported to experience pain, migraines, arrhythmia and blood pressure fluctuations [25], and plans should be in place to monitor and manage these.

Besides the available literature, a further important resource for metabolic specialists coordinating an MDT is national and international colleagues who are also responsible for metabolic patients. Conferences and meetings provide a further wealth of information across the field of MPS, which experts can use to inform up-to-date individualised care plans for each patient.

## Conclusions

The progressive heterogenous nature of MPS means that every adult patient is different, and management of critical clinical situations in MPS is based on being constantly aware of the potential for these to occur and establishing an individualised MDT who are prepared to respond to such events. An MPS expert is key to ensuring that all members of the team are supported with the relevant knowledge, and strategies are individualised to the patient. As patients with MPS are increasingly living longer, they are increasingly managed by adult care teams instead of paediatricians. Strategies for MPS will continue to evolve as patients survive into adulthood and adult care teams gain further experience of managing these complex disorders. Current clinicians managing patients with MPS should be aware of the potentially large number of symptoms and complications to be considered, but also that guidance and expertise is available to support them in optimising treatment plans for their patients.

## Methods

A group of experts including HCPs from centres with expertise in MPS from Germany, Spain, the Russian Federation and the UK met on two separate occasions to discuss transition management strategies for patients with MPS, and the management of adolescent and adult patients who have moved from paediatric to adult care. These centres all have experience of managing critical clinical situations in adults with MPS, and it was agreed that this expertise should be communicated to the healthcare community through a series of cases. These cases will provide examples of the types of factors that should be considered when preparing for interventions in patients with complex diseases, how paediatric specialists can be involved and how the wider MDT can prepare for potential challenges.

A series of teleconferences was organised to collect and discuss the required information and support publication development. Data and case studies were provided through written templates, telephone interviews and teleconferences between four contributing inherited metabolic disease centres in Europe:

- The Mark Holland Metabolic Unit, Salford Royal NHS Foundation Trust, Salford, United Kingdom
- Vall d’Hebron University Hospital, Barcelona, Spain
- HELIOS Dr. Horst Schmidt Kliniken Wiesbaden, Wiesbaden, Germany
- Research Center for Children’s Health, Moscow, Russian Federation

The eight cases presented highlight a selection of procedures that might be carried out in patients with MPS and have been carried out according to current protocols.

## Supplementary information

**Additional file 1.** Preoperative assessment.

## Data Availability

Not applicable as manuscript is based on case studies.

## Abbreviations

CT: Computed tomography
ECG: Electrocardiogram
ECHO: Echocardiogram
ENT: Ear, nose and throat
ERT: Enzyme replacement therapy
GAG: Glycosaminoglycan
HCP: Healthcare professional
MDT: Multidisciplinary team
MPS: Mucopolysaccharidosis
