# Supplementary material for: Critical clinical situations in adult patients with Mucopolysaccharidoses (MPS)
Source: Orphanet J Rare Dis. 2020 May 14;15:114. doi: 10.1186/s13023-020-01382-z (PMC7227065; doi:10.1186/s13023-020-01382-z)
Supplement: Supplementary file 1 — Additional file 1. Preoperative assessment. [file 13023_2020_1382_MOESM1_ESM.docx]

# Supplementary information

## Preoperative assessment

### Multidisciplinary review and MPS Passport template

The following information is collected by a multidisciplinary ‘one-stop clinic’ at the Salford Royal NHS Foundation Trust, Salford, UK.

This MPS Passport has been developed by the Department of Anaesthetics at the Salford Royal NHS Foundation Trust.

| **The MPS Passport** | |
| --- | --- |
| **Topic** | **Information collected** |
| **Reason for attendance** | - Regular assessment - Awaiting surgery - Awaiting IV ERT |
| **Diagnoses** | - MPS type - Other associated medical conditions |
| **Assessment** | - Age - Height - Weight - Body mass index - Facial appearance |
| **Intellectual ability** | - Learning difficulties - Special needs - Completion of consent forms for investigation or treatment or for adults lacking capacity to consent to investigation or treatment - Need for BIM |
| **Eyes** | - Corneal opacity - Visual acuity - Glaucoma - Retinopathy |
| **Head** | - Hydrocephalus - Headaches - Vomiting - Drowsiness |
| **Nose** | - Rhinorrhoea - Chronic ear infections |
| **Throat** | - Tonsillitis - Adenoid hypertrophy - Breathing difficulties |
| **Chest** | - Shape - Stiff rib cage - Pulmonary function tests - Increased risk of pulmonary infections |
| **Respiratory system** | - Noisy breathing - Snoring - Obstructive sleep apnoea - Need for CPAP - Secretion load - Difficulty clearing secretions - Asthma - Bronchitis - Frequent hospital admissions - Oxygen saturations at baseline and 20 m walk - Ear lobe oximetry |
| **Ears** | - Impaired hearing |
| **Oral cavity** | - Dentition - Enlarged tongue |
| **Cardiovascular system** | - Breathlessness - Valvular heart disease - ECG - Transthoracic ECHO - Transoesophageal ECHO |
| **Central nervous system** | - Epilepsy |
| **Gastrointestinal system** | - Nutrition and feeding issues - Endocrine issues - Hepatomegaly - Splenomegaly |
| **Abdomen** | - Hernias |
| **Bowel** | - Constipation - Diarrhoea |
| **Skin** | - Reduced elasticity - Increased thickness |
| **Bones and joints** | - Stiff joints - Dislocations - Contractures |
| **Spine** | - Gibbus - Scoliosis - Instability - Spinal cord compression |
| **Legs and feet** | - Deformities |
| **Hands** | - Deformities - Ease/difficulty of IV access - Carpal tunnel syndrome |
| **Carpal tunnel syndrome** | - Presence |
| **Investigations** | - Haematological   - Hb   - Full blood count   - Renal function   - Hepatic function   - Coagulation screen - Imaging   - X-ray cervical spine   - X-ray chest - CT or MRI of brain, neck (soft tissue) and/or chest - Urinary GAGs - ECG / 24-hour tape / ECHO - Carotid intimal thickness - EEG - Ear lobe oximetry - Standing and sitting blood pressure - Pulmonary function tests - Airway:   - CT/MRI scans   - 3D reconstruction   - 3D print of airway |

BIM, best interest meeting; CPAP, continuous positive airway pressure; CT, computed tomography; ECG, electrocardiogram; ECHO, echocardiogram; EEG, electroencephalogram; ERT, enzyme replacement therapy; GAG, glycosaminoglycan; Hb, haemoglobin; IV, intravenous; MRI, magnetic resonance imaging.

### Assessment of the airways and preparation for emergency tracheostomy

As well as carrying out the respiratory assessments described above in the multidisciplinary review, a more detailed assessment of the airway is also needed prior to surgery. This assessment should be carried out by two consultant anaesthetists, an ear, nose and throat (ENT) surgeon and an experienced operating department practitioner. Records of previous anaesthetic and airway procedures should also be examined, and any difficulties and how these were resolved should be noted.

| **Airway assessment** | - Bilateral patency of nasal cavities   - Assess ease of passing a fibre-optic scope for nasendoscopy and nasal intubation - Evidence of enlarged adenoids/tonsils - Evidence of acid reflux and need for a rapid sequence induction - Previous intubation and ventilation - Oral cavity   - Crowding of teeth   - Prominent teeth   - Mouth opening (Mallampati Grade) - Size of the tongue in relation to the oral cavity - Tongue mobility - Receding mandible - Mobility at cervical spine   - Flexion and extension   - X-ray flexion and extension views   - CT/MRI to rule out instability   - Spinal cord compression - Any abnormal deposits in the airway - Size and shape of the epiglottis / arytenoid cartilages / vocal cords, and the laryngeal inlet - Subglottic airway assessment   - By endoscopy under general anaesthetic   - By limited views from nasendoscopy   - Narrowing of or abnormalities in trachea-bronchial anatomy   - Length of the trachea from the vocal cords to the carinal bifurcation   - CT/MRI of the neck and thorax to assess anatomy and abnormalities of the trachea and bronchi, with review by a consultant radiologist   - 3D reconstruction of the airway   - 3D printing of the airway   - Trial intubation of 3D reconstruction     - For size of the bronchoscope     - For size and type of the endotracheal tube - Access to the front of the neck, in case of need for emergency tracheostomy   - Trachea centrally placed or deviated   - Ease of access to cricothyroid membrane   - Size of tracheostomy tube - Consent for:   - Failure to intubate   - Abandoning the procedure   - HDU or ICU admission for monitoring of airway and/or ventilation   - Controlled extubation |
| --- | --- |
| **Documentation of possible methods of and requirements for airway management / anaesthesia** | - Is IV access possible? - Is there a need for local anaesthetic cream or inhalational induction? - Awake/asleep endoscopy/intubation - Laryngeal mask airway   - Volume of upper airway GAG deposits - Endotracheal intubation   - Nasal/oral - Size of tubes   - Preparation of small tubes - Type of tube   - Microlaryngeal tube - Plan for secretion management - Choice of fibre-optic bronchoscopes - Rigid bronchoscope or Hopkins rod - Facilities to record pictures and live endoscopy videos for future reference - Choice of tracheostomy tubes - Plan for extubation - Role of IV steroids and endotracheal adrenaline prior to extubation - Extended recovery stay for monitoring - Need for:   - CPAP   - Transnasal humidified high-flow oxygen for apnoeic oxygenation - Postoperative destination   - General ward   - Monitored ward   - Critical care unit |

CPAP, continuous positive airway pressure; CT, computed tomography; GAG, glycosaminoglycans; HDU, high dependency unit; ICU, ﻿intensive care unit; IV, intravenous; MRI, magnetic resonance imaging.

Nasendoscopy and 3D airway reconstruction may be used to support assessment of the airways, if the patient consents to these procedures. Nasendoscopy can be carried out under a local anaesthetic, and the real-time video and image information collected may be more informative than CT and can be used to support individualised planning decisions for airway management by enabling greater understanding of any abnormalities present. Likewise, 3D airway reconstruction allows the multidisciplinary team to visualise abnormalities and practice different techniques to intubate the reconstructed airway prior to surgery. These models can also be used to explain symptoms and surgical challenges to patients and carers, and may be supportive in gaining informed consent for procedures, such as emergency tracheostomies. Patients with significantly deformed airways, severe spinal deformities, restricted access to the front of the neck or severe lung disease, and those undergoing prolonged procedures (especially in prone positions), may be more likely to require an emergency tracheostomy.

The anaesthetist, surgeons and patient should discuss the risk of an emergency tracheostomy and how the procedure would best be performed, and an experienced team with knowledge of managing difficult airways should be available to provide input. As physiological reserves are very limited in patients with MPS, the involvement of experienced clinicians is key to supporting optimal outcomes. The surgical team should also incorporate additional time into their plans for emergency procedures, such as tracheostomy for intubation difficulties or airway restrictions. A brief stay in an ICU for ventilatory support and gradual wean from ventilation is the most likely outcome for patients undergoing an emergency tracheostomy. This would be followed by a stay in the HDU and then a specialised ward so that the patient is safely weaned from ventilation and is provided with proper care for the tracheostomy. The possibility of a prolonged period of weaning until a decannulation is performed is very high, although discharge with a tracheostomy tube and home ventilation is possible.

### Discussion of procedures with the patient and family

The preoperative assessment provides an opportunity thorough discussion of the coming surgical procedure with the patient and their family. An understanding of the potential for an emergency tracheostomy is vital prior to using anaesthesia for either a diagnostic or a surgical procedure. Patients and carers should be provided with information on the likely length of stay in an HDU or ICU, the need for ventilation and the chances of needing a tracheostomy for a prolonged length of time with home ventilation. The palliative care team may be involved after surgery, based on discussions between the clinical team, the patient and their family.

## Surgical preparations

Drug and anaesthesia doses should be adapted for patients who have a smaller body surface area than that of an adult from the general population.

Prior to surgery, it should be ensured that the following are available and have been checked:

- Paediatric-sized cuffed and uncuffed endotracheal tubes
- Laryngoscopes of various sizes
- Laryngeal masks of various sizes and types
- Tracheostomy tubes of various sizes
- General anaesthesia equipment
  - A general anaesthetic is always required for a safe tracheostomy
- A standard difficult airway trolley as per guidance from the Difficult Airway Society
- Fibre-optic bronchoscopes and a rigid endoscope (Hopkins rod)

## Post-surgical care

Post-surgical care plans should be in place, including:

- A minimum 24-hour stay in post-surgical intensive care for all patients for observation
- Extended recovery stay
- Provision of CPAP / high-flow nasal humidified oxygen therapy
- HDU/ICU care for CPAP or ventilatory therapy
- A thorough post-tracheostomy care plan, encapsulating the following:
- An extended recovery stay, to identify and manage early complications
- A provision to support breathing with CPAP
- Transfer to HDU / ICU / a ward with nursing staff who have been specially trained to care for patients with a tracheostomy tube
- An assessment of the need for a period of ventilatory support on a case-by-case basis at the time of tracheostomy
- ERT plan
- Feeding plan
- In the immediate postoperative period, this will involve IV fluids
- Oral liquids and then solid food are allowed as tolerated
  - - The surgical procedure undertaken may delay initiation of oral feeding
    - A percutaneous endoscopic gastrostomy (PEG) tube, if already *in situ*, allows early commencement of PEG feeding to reduce the length of the fasting period
    - A PEG tube insertion may be inserted if the complex surgical procedure is expected to make swallowing difficult in the postoperative period
- Physiotherapy plan
  - A thorough preoperative assessment is required to inform the physiotherapy plan:
    - Risk factors such as chronic lung disease, secretion load and inability to cough should be identified
      - Physiotherapy is initiated preoperatively in patients with these risk factors
    - Earlobe blood gas assessment is a valuable investigation of respiratory function
    - An incentive spirometry is also helpful
    - Pulmonary function tests have limited value, as the normal values for patients with MPS are unknown
    - Frequency of chest infections should be assessed
    - If bronchospasm is detected, preoperative bronchodilators should be prescribed in the presence of any bronchospasm
    - Obstructive sleep apnoea should be identified, as patients have a high risk of postoperative apnoea, hypoxia and hypercarbia.
  - Physiotherapy should be started as soon as possible after the operation (even in the recovery area) and continued in the HDU/ICU environment
  - Early mobilisation is encouraged by nursing and physiotherapy staff
  - A daily clinical ward round should incorporate a physiotherapist
    - Plans to wean ventilation, make adjustments to tracheostomy tubes, or transfer to a ward should all be made in consultation with physiotherapists
  - Patients can be taken out of the ward in a wheelchair to improve their morale, which has a significant impact on their recovery
  - A step down to a specialised ward can enable patients to access uninterrupted physiotherapy
  - Discharge planning is undertaken with consultation of the physiotherapy and home ventilation teams if necessary
- Follow-up appointments in specialist outpatient clinics 6-weeks post-surgery to monitor recovery and rehabilitation
